# Where we should focus? Myths and misconceptions of long acting contraceptives in southern nations, nationalities and People’s region, Ethiopia: qualitative study

**DOI:** 10.1186/s12884-018-1731-3

**Published:** 2018-04-13

**Authors:** Misganu Endriyas, Akine Eshete, Emebet Mekonnen, Tebeje Misganaw, Mekonnen Shiferaw

**Affiliations:** 1 SNNPR Health Bureau, Hawassa, Ethiopia; 2 Department of Public Health, Dilla University, Dilla, Ethiopia

**Keywords:** Myths, Misconceptions, Contraceptive use, Method preference, Fear of side effects, SNNPR

## Abstract

**Background:**

Despite its wider benefits and access made at community level, contraceptive methods are one of underutilized services in study area and it is believed to be influenced by misconceptions and socio cultural values. This study was designed to explore women’s perceptions, myths and misconception to inform program implementers.

**Methods:**

Study was conducted in Southern Nations, Nationalities and People’s Region, Ethiopia in 2015. Five focus group discussions with 50 women of reproductive age and 10 key informant interviews with providers and program officers were done. The discussions and interviews were tape-recorded, transcribed verbatim and analyzed manually using framework analysis with deductive and descriptive approaches.

**Results:**

Improving community awareness about contraceptives and benefits of contraceptive utilization were acknowledged by majority of participants. Long acting methods were less preferred due to perceived side effects, myths and misconceptions and desire to have more children. Additionally, socio-economic status and partner influence were listed as reason for non-use. Poor provider-client interaction on available methods was also reported as system related gap.

**Conclusion:**

Program implementers need to address fears, myths and misconceptions. Quality of family planning counselling should be monitored.

## Background

Contraceptive utilization has multiple benefits; it can prevent unintended pregnancies, reduce the number of abortions, and lower the incidence of death and disability related to complications of pregnancy and childbirth. Increased contraceptive use and reduced unmet need for contraception are central to improving maternal health, reducing child mortality and also combating HIV/AIDS [1–3].

An estimated 225 million women in developing countries would like to delay or stop childbearing but are not using any method of contraception [3]. Ethiopia is one of the countries where significant progress has been recorded in family planning program in last decades. Contraceptive prevalence rate among married women was increased from 29% in 2011 to 42% in 2014 nationally while it was increased from 34% to 39.8% in Southern Nations, Nationalities and People’s Region (SNNPR) over the same period [4–6].

The fertility rate in developing counties including Ethiopia still remains high due to poor utilization of contraception [4–6]. Different studies showed that socioeconomic, demographic and psycho-social factors influence the utilization of contraception [7–13]. More specifically, women’s perception that they were not at risk of pregnancy, desire for birth spacing or limiting, ideal family size, lack of sufficient knowledge, religious and cultural reasons, culturally based gender inequalities and women’s previous experience of child death were reported as factors of utilization of contraception [4, 12, 14]. Fear of side-effects has been reported as an important hindering factor to contraceptive use especially in developing countries [15]. Studies showed that socio-cultural beliefs, myths and misinformation were negatively associated with contraceptive use [10, 11, 16].

In Ethiopia and study region, contraceptive methods are free and made accessible to household level through health extension program. The Ethiopian Ministry of Health had planned to increase contraceptive prevalence rate to 66% and couples’ approval of contraception use to 75% by the year 2015 [17] which was not achieved [18] nationally and in study region due to different reasons. Thus understanding women’s perceptions, myths and misconceptions was found key for program improvement.

## Method and materials

The study was conducted in SNNPR which is the third largest administrative region of Ethiopia representing about 20% of the country’s population. It is the most diverse region in the country in terms of language, culture and ethnic background. Administratively, the region is divided into 14 zones, 1 city administration and 4 special woredas (districts) [19]. Woreda is administrative structure (within zone or directly accountable to region if special) with approximate population of 100,000 population.

We used data collected for operational research to assess contraceptive utilization and associated factors in SNNPR Ethiopia in 2015 [20]. It was community based cross-sectional survey with mixed methods. First all woredas in region (regardless of zones they were located) were stratified in to urban, agrarian and pastoralist to represent regional variability of contraceptive utilization. Quantitative survey covered thirty three agrarian, five urban and two pastoralist woredas. Woredas for quantitative survey were selected using simple random sampling but for qualitative part, selection from survey sites was based on their low performance report focusing on challenges. Quantitative part of survey with limited qualitative support was written separately [20] and this is detail finding of qualitative part. We covered three agrarian, one urban and one pastoralist woreda, sample size depending on existing logistics and time [21] and size of each stratum in study region.

Five focus group discussions (FGDs) with women in reproductive age group were conducted. We included 10 discussants in each FGDs to have large enough range of different viewpoint and opinions. They were selected in consultation with health extension workers (government employed, community level public health workers). Participants’ nomination by health extension workers was based on their experience about community practice. FGDs discussants were leaders of one-to-five community development network and lead at least five households for more than two years. One-to-five women health development army (network) is community based experience sharing network for development purpose. In the network, one leader who better understands and implements health program packages leads neighbors, about five households. Team leaders teach community, address challenges they can and communicate with health extension workers (health care providers) on issues that are above their capacity.

As this study was operational, discussions focused on exploring barriers to inform program managers. The discussions took place in health posts and focused on their and community view on contraceptive methods, desire for more children, methods preference and reason for preference, fear of side effects and misconceptions about contraceptive utilization and possible solution/s to address challenges. All participants were encouraged to openly discuss their opinions and discussion continued until information was saturated; until discussion provided no new or relevant data [21].

Ten key informant interviews (KIIs) were also done; five with health extension workers /community level service providers/ and 5 with woreda (district) level program officers. Selection of KIIs was based on their working experience (above two years) in the area to express community experience and practice. The interview focused on plan achievements and challenges, community methods preference and reason for preference, fear of side effects and misconceptions about contraceptive utilization and possible solutions to challenges. Interviews took place in their offices (health post and district health offices) until information was saturated.

Interview and discussion guides were developed focusing on barriers of contraceptive utilization by exploring available literatures [4, 7, 9–16]. The developed guides were reviewed by research team and program experts if they could capture required data and for reducing ambiguity, leading questions, emotive questions and stressful questions thereby to address trustworthiness. Guides were prepared in English and translated into local language (Amharic) by investigators. Data were collected by investigators (male) who have master’s degree in public health and working on health research. Two investigators at a time (one interviewer, one note taker) conducted interviews and discussions. Each session lasted, on average, one hour.

After data collection, verbatim transcription of FGDs and KIIs were done and transcriptions were translated to English. Framework analysis [22–24] with deductive [24] and descriptive [25] approaches was used to summarize data as it is tailored for policy research and allows for inclusion of priori and emerging concepts [22–24]. Each of final transcription was read multiple times to validate transcription and familiarize with content and were compared with field notes. Preliminary themes were prepared by identifying priori storyline and categorizing data in to groupings. Key phrases and quotes were coded, classified and categorized repeatedly for deeper immersion. Preliminary themes were refined through subsequent revisions. Finally, groups of related data were clustered to similar themes. Quotes that best described main themes were chosen and presented.

Ethical clearance was obtained from the Ethical Review Committee of Regional Health Bureau. Informed verbal consent was approved considering rural and pastoralist women educational status. All participants gave verbal consent to participate after through explanation of benefits of study and all the information obtained was anonymous and kept confidential.

## Results

Fifty women of reproductive age, five health extension workers and five district level program officers were interviewed. All focus group discussants and health extension workers were female while program officers were male. Mean age of FGD discussant was 32.4 with standard deviation of 1.7 while mean age of HEW and program officers was 26.6 and 31.6 with standard deviation of 3.0 and 4.9 respectively. The FGD discussants participated in community health development network for 2–5 years (mean 3.5 ± 1.2). Working experience (in years) of HEWs and program officers ranged from 2 to 6 (mean 3.8 ± 1.5) and 4–14 (mean 7.2 ± 3.9) respectively.

The findings from both FGDs and KIIs were summarized in to the following three main themes.

### Attitude towards contraceptive methods and use

Majority of FGDs participants reported that family planning (FP) is essential for the health of mother and family. Except pastoralist participants who were not using contraceptive, majority of FGD participant said that family planning helped them to space children and improve productivity.

*“Long time ago people linked the use of FP with infidelity; but nowadays people know the importance of using FP and see it as a normal thing, it is not like in the past.” /30, Agrarian FGD participant/*

*“If we use FP, we would have fewer children and we can take care of them properly; providing better facilities, food and education to them.” (30, Agrarian FGD participants)*

All FGDs participants in pastoralist woreda were not using any FP methods. Their knowledge towards contraceptive methods was limited and they were not willing to use mainly because they wanted more children. Even after mentioning risks of delivering many children and associating it with women health, they did not believe.

*“I do not want to use contraceptive. I need more children. I would be happy if I could have 17 children.” (30, Pastoralist FGD participant)*

*“There is no risk for woman if woman eats good foods like meat of goat. What matters is what she eats. So, it is possible to have many children; we love many children.” (35, Pastoralist FGD participant)*

Even though FGD participants acknowledged benefits of contraceptive utilization, they raised some concerns related with contraceptive utilization, especially in rural areas where woman has limited power to decide. Some said that if they decide and use contraceptive without permission from partner, their partners relate any disease they face with contraceptive use and don’t provide medical cost. Rural participants also reported that some family prefer more children because more children are considered as labor for family.

*“We know that FP is good for our health but our husbands don’t let us to use. If we get sick, they say that sickness is due to contraceptive and don’t give us medical cost. So teach our husbands first.” (30, Agrarian FGD participants)*

Majority of service providers and woreda health officers said that their performance was below plan. They reported that behavioral, cultural and religious issues were major reason for their low performance.

“*The trends of contraceptive utilization was increasing from time to time but when we compared our performance with regional performance, we achieved low due to cultural influence and desire to have more children.” (35, pastoralist woreda program officer)*

*“In my community, having sex before marriage is taboo. Nobody is using condom because a person using condom is believed to be cheater. In addition, some educated people do not use contraceptives and tell us that they are using natural methods.” (25, agrarian service provider)*

*“About 90% of the population follows one religion and these people follow teachings that prohibit contraceptive utilization and we are performing below our plan.” (27, urban program officer)*

*“In our community, older women use contraceptive more than young women. This is because in our culture, women do not want to be pregnant when their daughters marry; in front of son-in-law”. (23, agrarian service provider)*

### Method of preferences

Majority of study participants agreed that due to the simplicity of the procedure, popularity by promotion and availability in health facilities, injectable was the most preferred method. They also said that injectable was highly preferred by young women, because it acts for short duration and it is easy to stop without seeking help from provider when they want to be pregnant. Long acting family planning methods were not preferred due to fear of side effect and misconceptions.

*“Since Depo-Provera was first promoted before introduction of long acting contraceptives, most clients had awareness and experience about it and use it.” /38, Agrarian program officer/*

*“Depo-Provera is preferred by young women because it acts only for three months and it is easy to stop without seeking help from provider when they want to be pregnant.” /31, Agrarian program officer/*

*“Most clients take contraceptive without the permission from their partners. For this reason, the simplest method preferred by clients is injectable as one cannot detect if a woman is using contraceptive easily.” /35, Pastoralist program officer/*

*“Implants are not preferred as women in the community believe that it is inserted by operation and think that it is difficult to stop since removal is not done at health post level. IUCD is also not preferred method in the community due to fear of the procedure of insertion.” /38, Agrarian program officer/*

Few FGDs participants reported that they were not familiar and clear with some of methods because providers did not explain sufficiently due to of shortage of consultation time. Some providers and program officers also forwarded such challenges related to quality of service provision. It was reported that when clients come to a health facility for the first time, the choice of contraceptives is not properly introduced by service providers; they simply give injectable and women continue using it. Some woreda program officers noted the skill gap of insertion and removal implants even after training.

*“When clients come to health facility for the first time, different choice of contraceptives were not introduced by service providers and they are not asked for their choice but Depo-Provera is simply given and women continue using it. I can say there is problem with some providers.” /26, Urban service provider/*

*“In addition to community preferences, we have gap of trained health extension workers on removal of implant, it is only done at health center level. Some health extension workers are still technically poor in providing even after training of insertion procedure.” /38, Agrarian program officer/*

### Fear of side effects and misconceptions

Even though community awareness and utilization was reported increasing over time, misconceptions and fear of contraceptive side effects in community were noted by study participants. Participants agreed that side effects mentioned were result of myths /misconceptions/. Misconceptions and fear of side effects mentioned ranged from less serious side effects (like weight gain) to association of death of woman with contraceptive utilization. Some rural participants associated contraceptive use with diet and refuse because they can’t get balanced diet. Commonly reported side effects included headache, disturbance of menstruations, weight gain, nausea, loss of appetite, infertility and other medical problems like hypertension, anemia, cancer, kidney stone and other disease after long use.

“*IUCD is not preferred method in our community due to fear of procedure. They believe that it may cause wound around their reproductive organ especially uterus and may even disappear after insertion during sexual intercourse.” (38, Agrarian program officer/*

*“My friend said if menstruation decreases or disappears, dirty blood would accumulate and can cause cancer.” (32, Agrarian FGD participant)*

*“I used injectable but I stopped using because I heard from my friend that this method can cause kidney disease, hypertension and other health disease. And also, one of my friend who was using injectable suffered over-bleeding and had irregular periods.” (32, Urban FGD participant)*

*“Some women said that implant moves to abdomen and it is not found in the insertion site. It moves to other body parts from the insertion site. It interferes with the routine activities.” (35, Agrarian FGD participant)*

*“I’ve heard from my friends that a woman using IUCD developed cancer. Finally, they linked cancer and death of that woman with contraceptive use.” (33, Agrarian FGD participant)*

*“Young women should not use contraceptives before their first pregnancy because if they use it, their uterus may become dirty and as a result they may not get pregnant.” /33, Agrarian FGD participant/*

*“The problem is getting balanced diet. Since, we don’t get balanced diet, we feel burning when we use it. Then we feel lightheadedness and start rounding.” /33, Agrarian FGD participant/*

## Discussion

Except pastoralist participants who were not using contraceptives, majority of FGDs participants agreed that contraceptive utilization is important for health of mothers and family, mentioning its benefit in limiting family size and spacing. Providers and program officers KIIs also supported that community awareness was improving. Majority of FGD participants preferred injectable method and was supported by interviews with providers and program officers. Besides this improvements, notable fears of side effects, myths and misconceptions were reported by participants. In addition, desire to have more children, religion, opposition from partner and lack of information on available methods were mentioned as challenges of contraceptive utilization.

The increasing community awareness about contraceptives, especially short term, has been reported by different studies in different countries including quantitative part of this study [12, 20, 26–28]. Moreover, short term contraceptives were preferred and utilized by majority of women in different studies [13, 14, 20, 27]. Even though reasons may vary across studies, in our study, long acting were not preferred due to lack of information, fear of side effects and myths and misconceptions. Such issues related to long acting contraceptives were also reported in different studies conducted in different areas [10, 15, 26–28].

Side effects reported in this study comprised anticipated method related side effects and fears and misconceptions they associated with contraceptive use. It included bleeding (disturbance of menstruation), headache, weight gain, nausea, loss of appetite and other medical disease such as hypertension, cancer, kidney stone and infertility. Beyond their perceptions, they also raised challenges they face from their partners. It was reported that even if women continue using contraceptives without partners’ consent, they face challenges from partners. They said that their partners also deny in the time of challenges like disease associating disease with contraceptive use. This challenge is a big problem for women in low-income country like our study area who have limited power to decide and have insufficient or no money to fulfil their needs. Spousal opposition has been reported by other study as reason for non-use [29].

Studies reported that myths, misinformation, and factual information about family planning were key predictors of contraceptive use [10, 16, 26]. And it was suggested that factual information encourages contraceptive use, while myths and misinformation discourages its use [10, 16]. We believe that in community like our study area where women have low awareness and limited access to information, community myths and misconceptions can contaminate health education easily as reported in other study [16]. For instance, an association of cancer and death due cancer with contraceptive utilization created fear among women as per FGD discussion. Such issues should be followed consciously and be addressed by concerned bodies, program implementers and/or providers.

In this study, we observed that women in pastoralist had limited knowledge even about benefit of contraceptives and available methods. What makes situation harder is belief that they reported “we don’t want to use it” justifying the need for more children.

Some FGD participants complained quality of counselling specifying inadequate information they get regarding side effects and options newly availed. They complained counselling (consultation) time was not adequate to communicate which was due to workload of providers. This issue and technical competency were also accepted by providers and program officers during interviews.

Lastly, study participants forwarded their views to improve utilization of family planning services. Some noted educating partners and improving provider-client interaction. Program officer from pastoralist area said the focus we gave to local leaders should be strengthened and we should use local leaders on posters to advertise contraceptives than using popular people elsewhere.

Even though this study could show key women’s perceptions and misconceptions, it is limited in addressing views of teens, men and religious leaders. In addition, though we believe that idea was saturated during discussions, there might be additional ideas in the region as the study region is the most diverse region in Ethiopia in terms of socio-cultural and ethnic background.

## Conclusion

Long acting were less preferred due to perceived side effects of contraceptives, myths and misconceptions. Poor provider-client interaction was noted as system related gap. Program implementers need to address misconceptions and quality of counselling should be monitored.

## Abbreviations

AIDS: Acquired Immune Deficiency Syndrome
FP: Family Planning
HIV: human immunodeficiency virus
IUCD: Intrauterine Contraceptive Device
SNNPR: Southern Nations, Nationalities, and People’s Region
